# Influence of the Novel Histamine H3 Receptor Antagonist/Inverse Agonist M39 on Gastroprotection and PGE2 Production Induced by (*R*)-Alpha-Methylhistamine in C57BL/6 Mice

**DOI:** 10.3389/fphar.2019.00966

**Published:** 2019-09-12

**Authors:** Salim M. A. Bastaki, Naheed Amir, Małgorzata Więcek, Katarzyna Kieć-Kononowicz, Bassem Sadek

**Affiliations:** ^1^Department of Pharmacology and Therapeutics, College of Medicine and Health Sciences, United Arab Emirates University, Al-Ain, United Arab Emirates; ^2^Department of Technology and Biotechnology of Drugs, Faculty of Pharmacy, Jagiellonian University-Medical College, Kraków, Poland

**Keywords:** H3 receptor, antagonist, M39, ulcer, C57BL/6 mice

## Abstract

The role of histamine H3 receptors (H3Rs) in the regulation of gastroprotection and production of prostaglandin E2 (PGE2) as well as somatostatin remains contradictory. Therefore, the effects of the H3R antagonist/inverse agonist M39 on *in vivo* acidified ethanol-induced gastric ulcers and gastric acid secretion in the C57BL/6 mice were assessed. Results showed that acute systemic administration of H3R agonist (*R*)-α-methylhistamine (RAMH, 100 mg/kg, i.g.) significantly reduced the severity of ulcer index, increased gastric acid output, and increased mucosal PGE2 production without any alteration of somatostatin concentration in gastric juice. However, only acute systemic administration of the H2R agonist dimaprit (DIM, 10 mg/kg, p.o.) significantly decreased the level of somatostatin measured in gastric juice. Moreover, acute systemic administration of M39 (0.3 mg/kg, i.g.) abrogated the RAMH-induced increase of acid output as well as PGE2 production, but not the DIM (10 mg/kg, i.g.)-stimulated acid secretion, indicating that RAMH as well as M39 modulate the gastroprotective effects through interactions with histamine H3Rs. The present findings indicate that agonistic interaction with H3Rs is profoundly involved in the maintenance of gastric mucosal integrity by modulating PGE2 as well as gastric acid secretion, with no apparent role in the regulation of the inhibitory influence of somatostatin.

## Introduction

Peptic ulcer is a chronic disease affecting up to 10% of the world’s population, and the formation of peptic ulcers depends on the presence of increased acidic gastric juice and the decreased mucosal defenses (Kuna et al., 2019). Non-steroidal anti-inflammatory drugs (NSAIDs) and *Helicobacter pylori* (*H. pylori*) infection are the two main factors disrupting the mucosal resistance to injury. Currently available conventional treatments of peptic ulcers include numerous pharmaceutical agents such as proton pump inhibitors, histamine H2-receptor antagonists, anticholinergics, antacids, antimicrobial agents, sucralfate, and bismuth, all of which are not fully effective, and are associated with numerous side effects including impotence, arrhythmia hematopoietic alterations hypersensitivity and gynecomastia (Chanda et al., 2011; Palle et al., 2018). Consequently, there is still a strong need for new pharmacologically active entities for the therapeutic management of peptic ulcer. Histamine exerts its biological activities through interaction with four distinct histamine receptors (H1R–H4R) that belong to the G-protein coupled receptor (GPCR) family (Lovenberg et al., 1999; Panula et al., 2015; Sadek and Stark, 2015; Sadek et al., 2016b). H1R and H2R are found in the brain and periphery. Although H4Rs are present in the brain, they are predominately expressed in mast cells and leucocytes, whereas H3Rs are abundant in CNS (Arrang et al., 1983; Arrang et al., 1985; Arrang et al., 1987a; Arrang et al., 1987b; Arrang et al., 1988; Arrang et al., 2007; Panula et al., 2015). H3Rs are coupled to G_i_/G_o_-proteins and act as auto-receptors that control the synthesis and release of histamine in the CNS, while activation of H1R and H2R mediates slow excitatory postsynaptic potentials (Arrang et al., 1983; Arrang et al., 1985; Arrang et al., 1987b; Sadek and Stark, 2015). Furthermore, H3Rs functioning as hetero-receptors can also regulate the release of other neurotransmitters like acetylcholine, glutamate, GABA, norepinephrine, serotonin, dopamine in variable brain regions (Brown et al., 2001). Mounting preclinical experimental evidences related numerous functional and behavioral effects to central H3R-mediation. The activation of such cerebral sites appears to stimulate a waking effect in cats (Schwartz et al., 2003; Lin et al., 2008; Lin et al., 2011), modulation of locomotor activity, anticonvulsant, antinociceptive, and procognitive actions in mice and rats (Clapham and Kilpatrick, 1993; Yokoyama et al., 1993; Clapham and Kilpatrick, 1994; Malmberg-Aiello et al., 2003; Sadek et al., 2013; Sadek et al., 2014a; Sadek et al., 2014b; Sadek et al., 2015; Sadek and Stark, 2015; Sadek et al., 2016a; Sadek et al., 2016b; Sadek et al., 2016c). Interestingly, several polymorphisms have been detected for H3Rs (Hancock et al., 2003; Bongers et al., 2007), and H3Rs were found to be expressed in dimers (Shenton et al., 2005; Bakker et al., 2006) as well as co-expressed with other GPCRs, e.g. dopamine D1- or D2 receptors and adenosine A2A receptors in form of heteromers, and were linked with their modulating effects on several brain neurotransmitters, and therefore, their influence in mitigating numerous brain disorders (Ferrada et al., 2008; Ferrada et al., 2009; Moreno et al., 2011; Rodriguez-Ruiz et al., 2017; Marquez-Gomez et al., 2018). Notably, complexity of histamine H3R biology e.g. many isoforms, constitutive activity, the aforementioned heteromerization with other receptors (dopamine D2, D1, adenosine A2A), and pharmacology make it difficult to realize and evaluate the therapeutic potential of H3R antagonists (Lazewska and Kiec-Kononowicz, 2018).

Moreover, H3R has been identified in the gastrointestinal tract of the rat by immunohistochemistry, as immunoreactivity to H3R was exclusively localized to the endocrine cells scattered in the gastrointestinal mucosa, with positive cells being prominently abundant in the gastric fundus, while they were rarely found in the other regions (Grandi et al., 2008). 

Contrary, peripheral administration of the selective H3R agonist (*R*)-α-methylhistamine (RAMH) was found to inhibit pentagastrin-, 2-deoxy-D-glucose-, and peptone meal-stimulated gastric acid secretion in conscious cats as well as pentagastrin- and bombesin-induced hypersecretion in the conscious dog (Bado et al., 1991; Soldani et al., 1993). The latter inhibitory effects were also found to be antagonized by co-administration of the H3R antagonist thioperamide (Soldani et al., 1993). Moreover, numerous previous *in-vitro* experimental models of gastric injury showed that H3R agonist RAMH exerts protective effects with controversial interpretations (Morini et al., 1995a; Morini et al., 1995b; Morini et al., 1997; Morini et al., 2008b). For instance, RAMH was likely to inhibit acid secretion via the suppression of histamine in isolated rat fundic enterochromafin-like cells, whereas an increased acid secretion secondary to reduced somatostatin secretion is reported in isolated mouse stomach (Morini et al., 1995a; Morini et al., 1995b; Morini et al., 1997; Morini et al., 2008b). Furthermore, early *in-vivo* experiments showed that RAMH also potently inhibits gastric secretion by a number of indirect stimuli, e.g. vagal stimulation or pentagastrin (Bado et al., 1991; Yokotani et al., 2000). In addition, the role of H3Rs in the gastric mucosal gastrin expression and release by inhibiting secretion of somatostatin is still unclear, since the mechanism of action underlying this phenomenon remains unknown and is still a matter of study even though the presence of H3Rs on parasympathetic nerve terminals or on gastric paracrine cells has been proposed (Soll and Walsh, 1979; Bado et al., 1991; Soldani et al., 1993; Soldani et al., 1994; Coruzzi et al., 2001). In addition, unclear data were obtained with two highly selective H3R agonists, namely imetit (Garbarg et al., 1992) and immepip (Vollinga et al., 1994), which failed to share the gastroprotective effect of RAMH towards 0.6 N HCl-induced gastric damage in rat. However, it is well-known that many imidazole-containing ligands, including immepip and imetit, display affinity for the H4R (Lim et al., 2005), indicating that it might be postulated that histamine H4 receptor-mediated mechanisms may have influenced the interpretation of the observed results for immepip and imetit (Coruzzi et al., 2011). The H4R was primarily identified on immunocompetent cells and cells of the hematopoietic lineage, such as mast cells, eosinophils, basophils, dendritic cells and T cells and a primary role in the inflammatory responses was postulated (Leurs et al., 2009). Therefore, H4R antagonists are under development as novel antiallergic and anti-inflammatory drugs (Thurmond et al., 2008; Zampeli and Tiligada, 2009; Sadek and Stark, 2015). Additionally, H4R expression was detected by immunohistochemistry in different areas of the gut (Cianchi et al., 2005; Sander et al., 2006; Breunig et al., 2007; Boer et al., 2008; Morini et al., 2008a) and protective effects have been evidenced, by the use of the H4R antagonist JNJ7777120 in various rodent models of gastric and intestinal damage (Thurmond et al., 2004; Varga et al., 2005; Coruzzi et al., 2007), demonstrating the involvement of the H4Rs in gastrointestinal inflammation and ulcerogenesis (Coruzzi et al., 2012).

**Figure 1:**
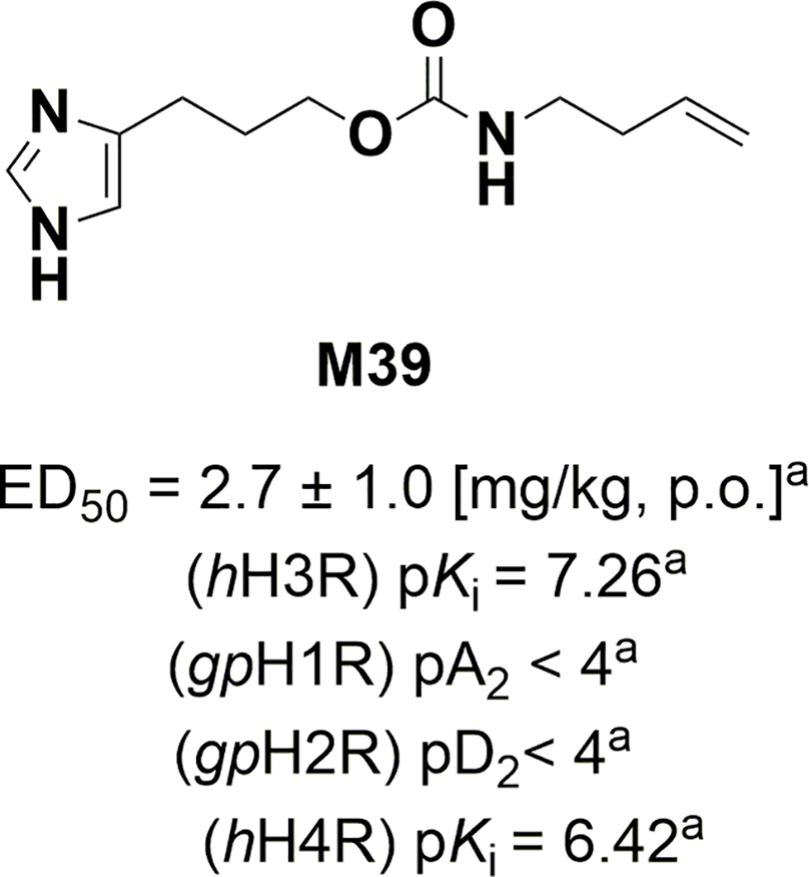
Structure, *in-vivo* potency, and *in-vitro* antagonist affinities of H3R antagonist/inverse M39. ^a^Values previously published (Wiecek et al., 2011; Sadek et al., 2013). Values for H1R and H2R were tested on guinea pig (gp).

 To date, the potential contribution of a central H3R-mediated *in-vivo* regulatory influence in affecting gastric acid secretion has not been extensively explored and as a result cannot be ruled out completely. The latter considerations together with the aforementioned controversies in results obtained so far encouraged us to investigate the possible involvement of H3Rs in the control of gastric secretory function. Therefore, the objective of the current study was to determine whether interactions with H3Rs mediate *in vivo* gastroprotection applying the acidified ethanol-induced gastric ulcers and gastric acid secretion model in C57BL/6 mice, and following intragastric (i.g.) administration of the potent and selective H3R antagonist/inverse agonist M39 in presence and absence of the selective and potent H3R agonist RAMH (**Figure 1**). Furthermore, the modulating effects of both H3R compounds on synthesis of somatostatin as well the production of prostaglandin E2 (PGE2) were investigated.

## Material and Methods

### Animals

C57BL/6 mice were purchased from Jackson Laboratory, Boulevard, Bethesda, MD 20892-4874, USA. They were bred at our animal facility. Adult male C57BL/6 mice (14–15 weeks) weighing 25–28 g were fasted for 24 h in wire mesh cages to avoid coprophagy but had free access to water ad libitum. The temperature of the animal room were maintained at 22 ± 2°C and with a 12–12 h dark–light cycle. This study was carried out in accordance with the recommendations of the European Communities Council Directive of 24 November 1986 (86/609/EEC), and was approved by the Institutional Animal Ethics Committee in the College of Medicine and Health Sciences/United Arab Emirates (Approval No. A38-13). All efforts were made to minimize animal suffering and to reduce the number of animals used.

### Chemicals and Drugs

The H3R antagonist/inverse agonist 3-(1*H*-imidazol-4-yl)propyl but-3-en-1-ylcarbamate (M39) was synthesized by us in the Department of Technology and Biotechnology of Drugs (Kraków, Poland) as described previously (Wiecek et al., 2011) (**Figure 1**). The compound M39 was synthesized by a modified Curtius reaction. In this reaction 4-pentenoic acid was reacted with diphenyl phosphorazidate (DPPA) under basic conditions leading to in situ obtained isocyanate intermediate, which then was reacted with 3-(1*H*-imidazol-yl)propanol hydrochloride to yield M39 (Wiecek et al., 2011).

All other drugs and chemicals were purchased from Sigma-Aldrich (St Louis, Missouri, USA). All drug solutions were freshly prepared. The enzyme immunoassay kit of Somatostatin was purchased from Phoenix Pharmaceuticals, Inc., Burlingame, CA 94010, USA. Enzyme immunoassay kit of PGE2 was purchased from R&D Systems, Minneapolis, MN, USA. 

### Gastric Acid Secretion *In Vivo*

Mice were initially anaesthetized with pentobarbitone (70 mg/kg) intraperitoneally (i.p.). Following general anesthesia, the pylorus was ligated according to the method of Shay et al, 1945 (Shay et al., 1945; Wiecek et al., 2011). Briefly, after the ligation, the animals were assigned into six different groups of six mice as follows: group 1: control, pretreated with distilled water (DW), group 2: RAMH (100 mg/kg, i.g.), group 3: H2R agonist DIM (10 mg/kg, i.g.), group 4: M39 (0.3 mg/kg, i.g.), group 5: M39 (0.3 mg/kg, i.g.)+RAMH (100 mg/kg, i.g.), and group 6: M39 (0.3 mg/kg, i.g.)+DIM (10 mg/kg, i.g.).

All the drug solutions were administered to the animals by gastric gavage. However, both groups 5 and 6 received H3R antagonist/inverse agonist M39 (0.3 mg/kg) followed by 15 min later administration of RAMH or DIM, respectively. The dose of 0.3 mg/kg of M39 was selected based on a dose response (0.3–3.0 mg/kg) pilot study as well as according to previous study conducted in Wistar rats (Morini et al., 2000). After 4 h, the animals were sacrificed by cervical dislocation and the gastric juice was collected for somatostatin assay and gastric contents were titrated against 0.01M sodium hydroxide for acid output which was calculated and expressed as mmol/4h.

### Assay of Somatostatin in Gastric Juice

Competitive Enzyme immunoassay of somatostatin in gastric juice was performed according to manufacturer’s protocol. Briefly, the immunoplate was pre-coated with secondary antibody and the nonspecific binding sites were blocked. The secondary antibody was allowed to bind to the Fc fragment of the primary antibody whose Fab fragment was competitively bound by both biotinylated peptide and peptide standard or targeted peptide in samples during 2 incubation at room temperature (20–23°C) on orbital shaker at 300–400 rpm. The interaction of biotinylated peptide with streptavidin–horseradish peroxidase (SA–HRP) was catalyzed by the substrate solution. The intensity of the color is directly proportional to the amount of biotinylated peptide– SA–HRP complex but inversely proportional to the amount of the peptide in standard solutions or samples. The color intensity was read at 450 nm with a microplate reader (Tecan Group Ltd., Männedorf, Switzerland). Somatostatin concentration was expressed as nanogram per ml of gastric juice.

### Gastric Acid Ulcer *In Vivo*

Acidified ethanol (AE; 60% ethanol in 150 mM hydrochloric acid) was used to induce gastric ulcers in the mice. The mice were divided into four different groups of 6 animals each. Each group received either DW (control), or RAMH (100 mg kg), or M39 (0.3 mg/kg) 15 min prior to RAMH (100 mg/kg) administration as a gastric gavage. After 30 min of DW or drug administration, AE was given orally to each animal at a dose of 0.2 ml per mouse and the animals were killed 1 h later by cervical dislocation. The abdomen was incised and the stomach removed. The stomach was cut open along the greater curvature and rinsed with saline to remove any adherent food particles and mucus. The opened stomach was then spread on a sheet of cork so as to have a clear macroscopic view of the gastric mucosa. The total lengths of the haemorrhagic lesions, which were approximately 1 mm in width and formed in the glandular portion of the gastric mucosa, were taken as ulcer index (UI). An observer unaware of the drug treatments confirmed the ulcer index. The use of 60% ethanol in 150 mM HCl as an ulcerogenic agent was based on our earlier observation and of others that ethanol 50% and over provided a reproducible model of gastric damage (Nishida et al., 1994; Mercer et al., 1995; Chandranath et al., 2002; Bastaki et al., 2003).

#### Preparation of Gastric Mucosal Homogenate

Briefly, after dissection, stomachs were washed with ice-cold PBS, and the gastric mucosa was rapidly scraped from the underlying tissue layers of stomach on ice. The mucosa was weighed, minced by forceps, and homogenized with 3 volumes of cold phosphate buffer (PBS 0.1 mol/L, pH 7.4, containing 1 mM EDTA and 10 μM indomethacin) per gram of tissue using a polytron homogenizer (IKA laboratory, Germany).

### Assay of PGE2 in Gastric Mucosa

Competitive Enzyme immunoassay of PGE2 in gastric mucosal was performed according to manufacturer’s protocol. This assay was based on the forward sequential competitive binding technique in which PGE2 present in a sample competed with horseradish peroxidase (HRP)-labeled PGE2 for a limited number of binding sites on a mouse monoclonal antibody. PGE2 in the sample was allowed to bind to the antibody in the first incubation of one hour at room temperature on a horizontal orbital microplate shaker (0.12” orbit) at 500 ± 50 rpm. During the second incubation, HRP-labeled PGE_2_ was bound to the remaining antibody sites. Unbound materials were washed and substrate solution was added to the wells to determine the bound enzyme activity. The color development was stopped, and the absorbance was read at 450 nm. The intensity of the color was inversely proportional to the concentration of PGE2 in the sample. PGE2 concentration was expressed as picogram per milligram of mucosal tissue.

### Statistical Analysis

Data was analyzed statistically using SPSS 25.0 software (IBM Middle East, Dubai, UAE). The means of the data are presented with the standard error mean (SEM). The results were analyzed using independent *t*-test to determine the significance of the mean between the groups. Values of *P* < 0.05 were considered significant.

## Results

### Acidified Ethanol-Induced Gastric Mucosal Lesions

#### Macroscopy

**Figure 2:**
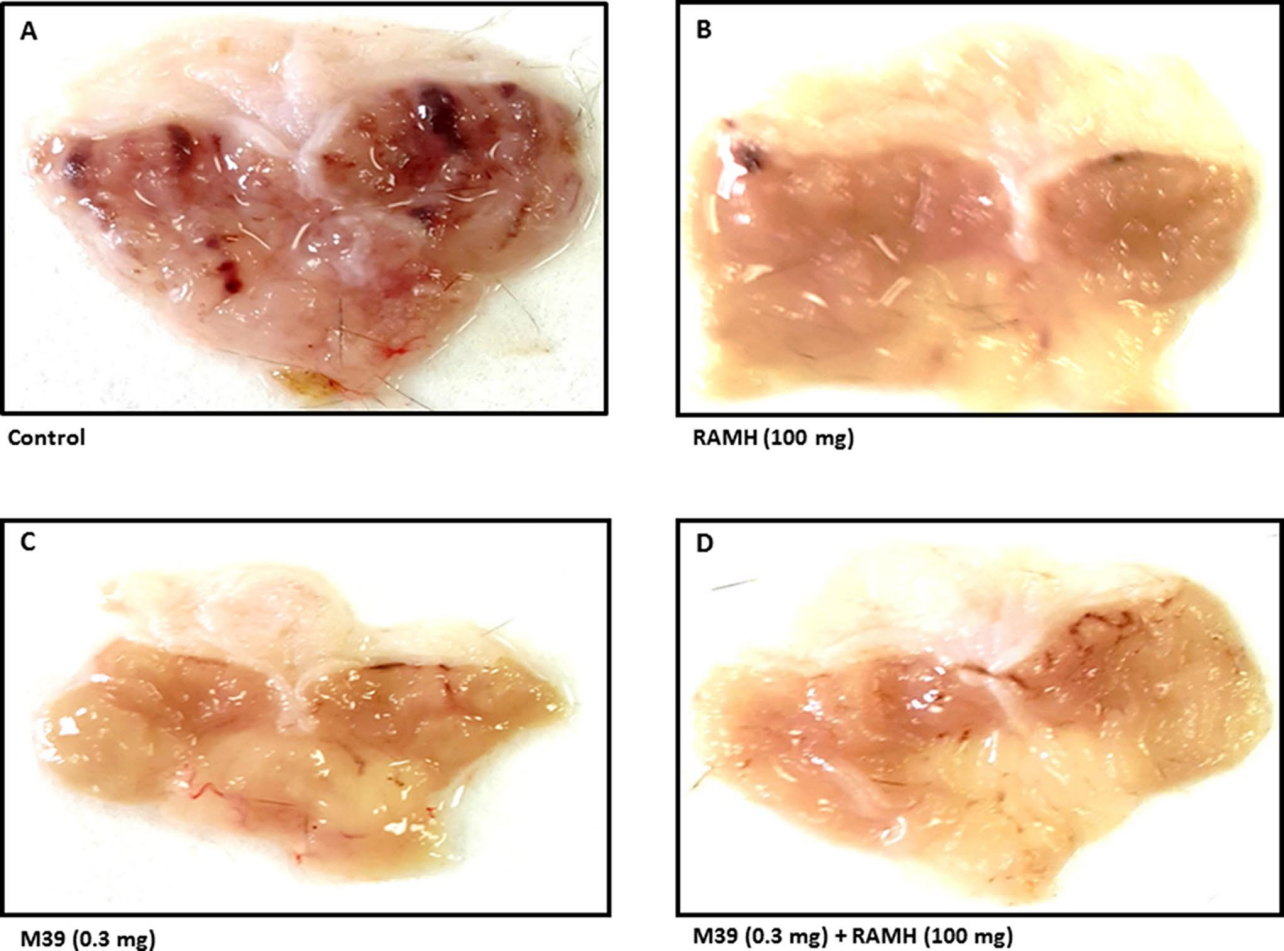
Photomicrographs of stomachs showing the effect of vehicle, H3R agonist (*R*)-α-methylhistamine, and test compound M39 on stomach ulcer of C57BL/6 mice. Micrographs are the results of five to six such experiments. showing the effect on acidified ethanol in **(A)** vehicle-, **(B)** RAMH(100mg/kg)-, **(C)** M39(0.3mg)-, and **(D)** M39(0.3mg)+RAMH(100mg)-treated group in reducing the stomach ulcers.

**Figure 3:**
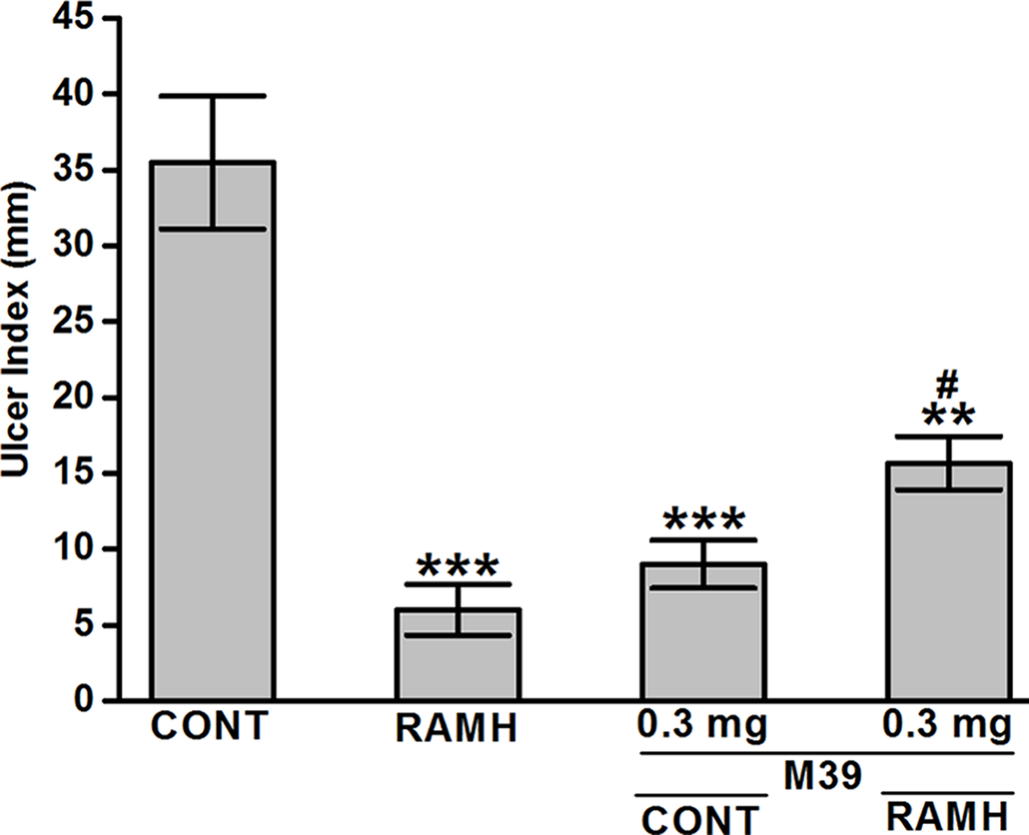
Effects of vehicle, H3R agonist (*R*)-α-methylhistamine, and test compound M39 on acidified ethanol-induced ulcer index in C57BL/6 mice. **P < 0.01 vs. vehicle-treated group. ***P < 0.001 vs. vehicle-treated group. ^#^ *P* < 0.05 vs. RAMH- or M39(0.3mg)-treated group. Data represent mean ± SEM (n = 5–6).

Acidified ethanol determined the formation of red to black linear streaks in the glandular portion of the stomach of vehicle-treated group (ulcer index of 35.5 ± 4, n=6) (**Figures 2** and **3**). Mucosal damage caused by acidified ethanol was substantially reduced by RAMH at the dose of 100 mg/kg (ulcer index of 6.0 ± 1.46, n = 6; *P* < 0.001). Similarly, M39 when administered alone at a dose of 0.3 mg/kg significantly reduced the mucosal damage (ulcer index of 9.0 ± 1.41, n = 6; *P* < 0.001) (**Figures 2** and **3**). However, RAMH and M39 (0.3 mg/kg) failed to completely abrogate each other’s protections when compared to vehicle-, RAMH(100mg/kg)-, M39(0.3mg)-group with ulcer index of 15.67 ± 1.44, n = 6; both *P* < 0.05 for RAMH(100mg)+M39(0.3mg)-treated group versus RAMH- or M39(0.3mg)-treated group) (**Figures 2** and **3**).

#### PGE2 Synthesis

**Figure 4:**
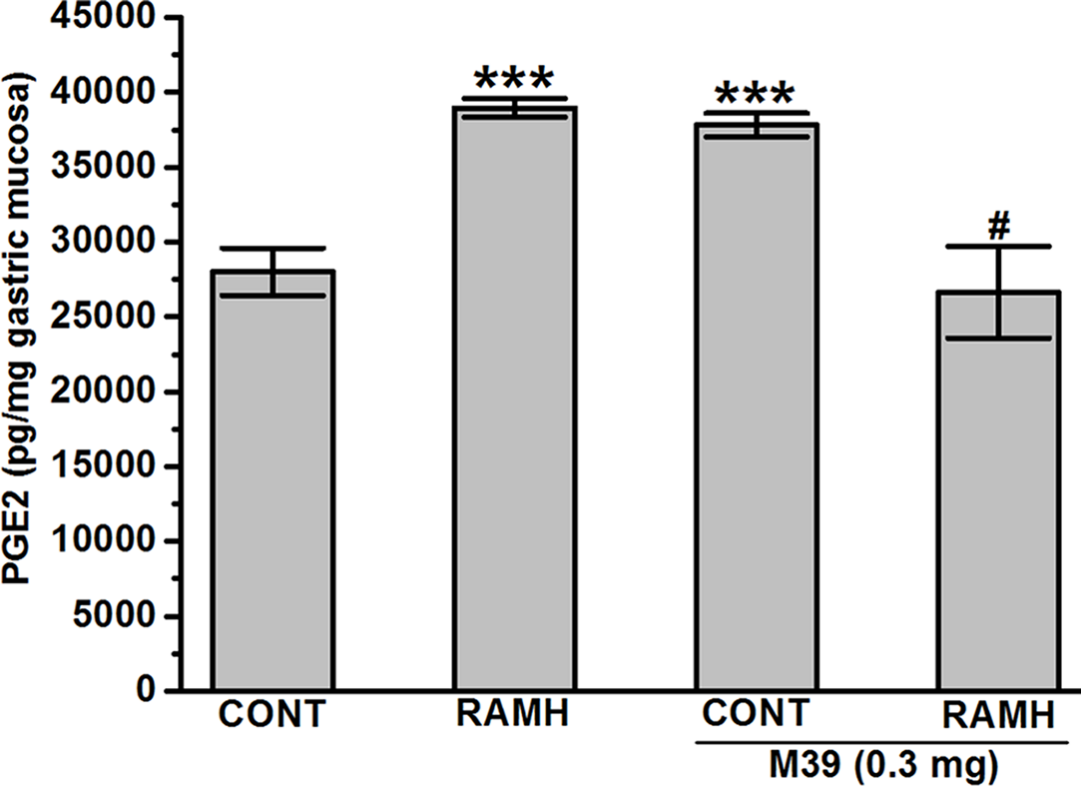
Effect of vehicle, H3R agonist (*R*)-α-methylhistamine, and test compound M39 on PGE2 synthesis in the stomach mucosa of C57BL/6 mice. ***P < 0.001 vs. vehicle-treated group. ^#^ *P* < 0.05 vs. RAMH- or M39(0.3mg)-treated group. Results are expressed as the mean ± standard error of six replicates.

The observed results show that both the H3R agonist RAMH (100 mg/kg) and M39 (0.3 mg/kg) administered separately significantly increased the synthesis of mucosal PGE2 with values of 38971.17 ± 624.44 and 37828.98 ± 782.45 pg/mg of gastric mucosa, respectively (*P* > 0.001 for RAMH- and M39-treated group versus vehicle-treated group), respectively. However, co-administration of both M39 and RAMH abrogated each other’s stimulating effect on PGE2 synthesis with a value of 26650.72 ± 3068.16 pg/mg (p = 0.65 for M39+RAMH-treated group versus vehicle-treated group) (**Figure 4**).

### Somatostatin Synthesis

**Figure 5:**
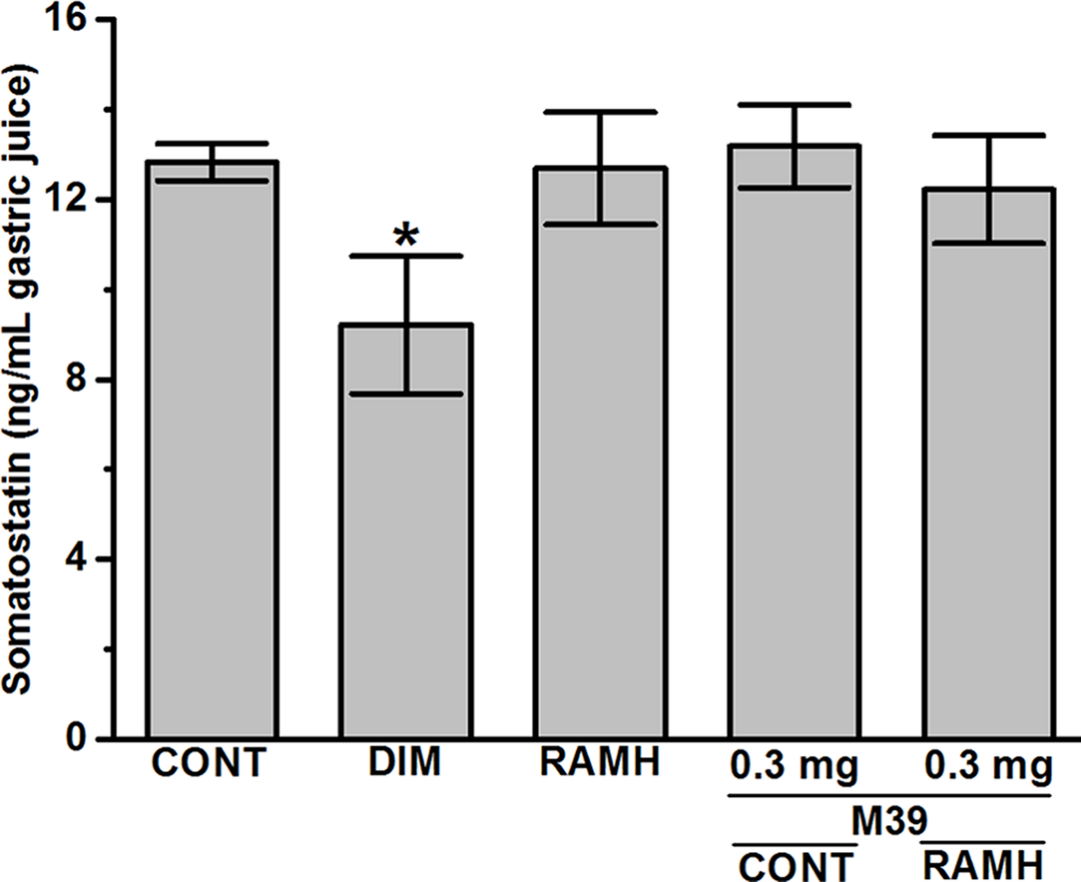
Effects of vehicle, H2R agonist dimaprit, H3R agonist (*R*)-α-methylhistamine, and test compound M39 on gastric somatostatin concentration in C57BL/6 mice. Data represent mean ±SEM (n = 5–6). *P < 0.05 vs. vehicle-treated group.

The results observed show that only DIM significantly decreased the synthesis of somatostatin (12.83 ± 0.36 ng/mL for vehicle-treated group versus 9.21 ± 1.25 ng/mL for DIM-treated group; *P* < 0.05). However, RAMH and M39 (0.3 and 3 mg) failed to modulate the biosynthesis of somatostatin with values of 12.47 ± 1.33, 13.18 ± 0.80, and 11.21 ± 1.10, respectively (all *P* values > 0.05) (**Figure 5**).

### Gastric Acid Secretion

**Figure 6:**
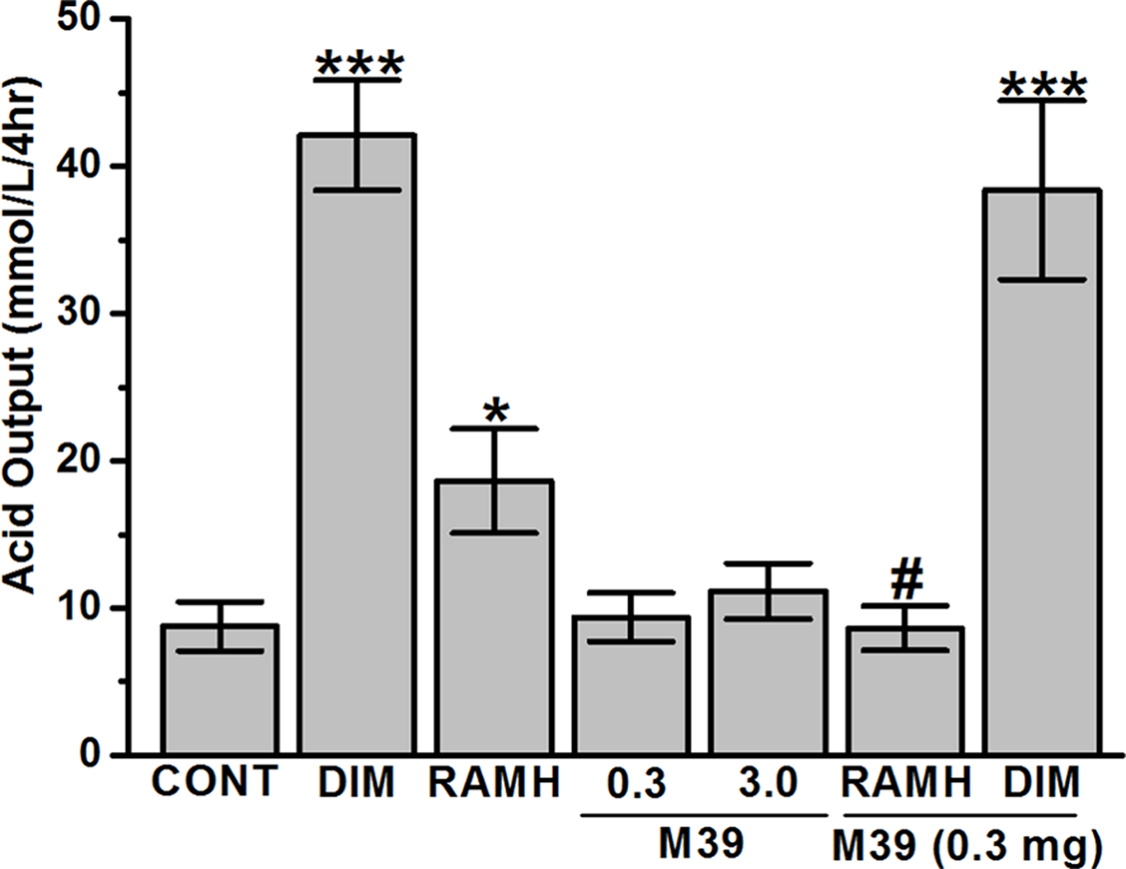
Effects of vehicle, H2R agonist dimaprit, H3R agonist (*R*)-α-methylhistamine, and test compound M39 on gastric acid secretion in the stomachs of C57BL/6 mice. Data represent mean ±SEM (n = 5–6). *P < 0.05 vs. vehicle-treated group. ***P < 0.001 vs. vehicle-treated group. ^#^ *P* < 0.05 vs. RAMH-treated group.

In the juice present in the stomach, the H2R agonist DIM and the H3R agonist RAMH caused a significant increase in the volume of gastric juice with a concomitant marked and significant increase in the amount of titratable acidity (**Figure 6**). The stimulation of acid secretion by RAMH, was completely reversed by prior treatment with the H3R antagonist/inverse agonist M39 (p = 0.96 for vehicle-treated group versus RAMH+M39(0.3mg)-treated group) (**Figure 6**). However, M39 (0.3 mg/kg) failed to reverse the effect observed by DIM (p = 0.63 for DIM-treated group versus DIM+M39(0.3mg)-treated group) (**Figure 6**). In addition, H3R antagonist/inverse agonist M39 (0.3 and 3 mg/kg) had no effect on titratable acidity when administered alone as compared to vehicle-treated group (p= 0.36 and p= 0.36, for M39(0.3mg)-treated group versus vehicle-treated group and M39(3mg)-treated group versus vehicle-treated group, respectively) (**Figure 6**).

## Discussion

The current study indicates that RAMH efficiently preserves the integrity of mice gastric mucosa against damage with acidified ethanol, as measured using macroscopic evaluation (**Figure 2**). In the current *in-vivo* gastric ulcer model, the H3R agonist RAMH and the H3R antagonist/inverse agonist M39 exhibited comparable gastroprotective effects as obviously observed by ulcer indices (**Figure 3**). Moreover, the results observed show that the gastroprotective effect provided by H3R agonist RAMH was partly reversed when mice were pretreated with M39 as measured on the level of ulcer index, indicating that, in addition to histaminergic pathways through activation of H3Rs, the protective effects might be attributed to mechanisms other than histaminergic neurotransmission. These results are in agreement with a previous study which showed that H3R is involved in the protection of rat stomach against concentrated hydrochloric acid. However, the functional role of the H4R is still to be defined, although selective agonists induce proulcerogenic effects under hydrochloric acid challenge (Coruzzi et al., 2011). Furthermore, both compounds when administered separately significantly increased the level of synthesized PGE2, an effect that might explain the gastroprotection provided by both compounds (**Figure 4**). Interestingly, the stimulating effect observed for RAMH on PGE2 synthesis was completely abolished when mice were pretreated with the potent H3R antagonist M39 (**Figure 4**). However, the H3R agonist RAMH as well as the H3R antagonist/inverse agonist M39 failed in the current study to modify the synthesis of somatostatin (**Figure 5**). The latter results are in disagreement with previous studies in which modulation of H3Rs resulted in a significant alteration of somatostatin synthesis, demonstrating that the mechanisms responsible for the protective action observed for RAMH and H3R antagonist/inverse agonist M39 appear to be unrelated to their effects on somatostatin synthesis (Morini et al., 1995a; Morini et al., 1995b; Morini et al., 1997; Morini et al., 2000). Moreover, protection by RAMH is not attributable to a modulation in gastric acidity, since RAMH increased acid production and secretion, although significantly lower than the effect observed for the H2R agonist DIM (**Figure 6**). Furthermore, the H3R antagonist/inverse agonist M39 failed to modify gastric acid secretion which is in agreement with previous studies in which ciproxifan was found to be not effective in increasing or decreasing gastric acid secretion (Morini et al., 2000). Interestingly, the effect of RAMH, but not of DIM, on increasing gastric acid secretion was completely abrogated when mice were pretreated with M39 (**Figure 6**). These observations clearly indicate that there is no association between the effects observed for RAMH and M39 on gastric acid secretion and their influence on the susceptibility of the mucosa to lesion formation. Notably, there is still some debate as to whether the stimulation of gastric acid secretion evoked by RAMH in rodents is attributable to H3R activation (Vuyyuru and Schubert, 1997; Coruzzi and Bertaccini, 1998). However, the present failure of H3R antagonist/inverse agonist M39 to influence RAMH-stimulated acid secretion at doses causing a complete inhibition of RAMH-induced increase of PGE2 synthesis appears to rule out the involvement of H3Rs in the acid secretory response to RAMH. Therefore, caution is required when interpreting the effects observed for H3R agonists or antagonists/inverse agonists in preclinical ulcer models. Accordingly, the differences in animal species together with the type of experimental conduct and the routes by which the test compounds are administered should be considered for comparison when interpreting results observed. Additionally, it should be taken into consideration that RAMH and M39 are likely to differ significantly in their pharmacokinetic properties when it comes to testing them in *in-vivo* models.

## Conclusion

The partial M39-provided inhibition of the gastroprotection exerted by RAMH validate the hypothesis that RAMH is capable of counteracting mucosal damage mediated through histamine H3Rs. However, here we present the first evidence that agonistic interaction with H3Rs are actively involved in the maintenance of gastric mucosal integrity by modulating PGE2 as well as gastric acid secretion, with no apparent role in the regulation of inhibitory influence of somatostatin. However, the observed results showed that M39 reverses the effects of RAMH on gastroprotection and PGE2 production, demonstrating that it is still difficult to provide a conclusion since both compounds were able to show effects on their own, and combination of both counteracts the effects of either alone. Therefore, the exact mechanistic background for the mediated protective effects of RAMH as well as M39 still continues to be a challenge.

## Data Availability

The raw data supporting the conclusions of this manuscript will be made available by the authors, without undue reservation, to any qualified researcher.

## Author Contributions

BS and SAB were responsible for the study concept, design, and acquisition and analysis of data. NA conducted all experiments. KK-K and MW were responsible for the generation, synthesis, and pharmacological *in vitro* characterization of the test compound M39. BS and SAB drafted the manuscript. KK-K, MW, and NA critically revised the manuscript. All authors critically reviewed content and approved the final version of the manuscript for publication.

## Funding

SB and BS were supported by intramural funds from the College of Medicine and Health Sciences and the Office of Graduate Studies and Research, UAE University. The authors acknowledge the partial support of Jagiellonian University statutory funds (K/ZDS/007121) and EU COST Action MuTaLig CA15135 to KK-K and MW.

## Conflict of Interest Statement

The authors declare that the research was conducted in the absence of any commercial or financial relationships that could be construed as a potential conflict of interest.
